# Chitosan Stabilized Silver Nanoparticles for the Electrochemical Detection of Lipopolysaccharide: A Facile Biosensing Approach for Gram-Negative Bacteria

**DOI:** 10.3390/mi11040413

**Published:** 2020-04-14

**Authors:** Muhammad Imran, Christopher J. Ehrhardt, Massimo F. Bertino, Muhammad R. Shah, Vamsi K. Yadavalli

**Affiliations:** 1 Department of Chemical & Life Science Engineering, Virginia Commonwealth University, Richmond, VA 23284, USA; imranbjr.khan@gmail.com; 2 H.E.J. Research Institute of Chemistry, International Center for Chemical and Biological Sciences (ICCBS), University of Karachi, Karachi 75270, Pakistan; raza.shah@iccs.edu; 3 Department of Forensic Science, Virginia Commonwealth University, Richmond, VA 23284, USA; cehrhardt@vcu.edu; 4 Department of Physics, Virginia Commonwealth University, Richmond, VA 23284, USA; mfbertino@vcu.edu

**Keywords:** chitosan, silver nanoparticles, lipopolysaccharide, *Escherichia coli*, biosensor

## Abstract

Negatively charged lipopolysaccharide (LPS), a major endotoxin and component of the outer membrane of several Gram-negative bacteria, provides a useful biomarker for the indirect detection of these pathogens. For instance, *Escherichia coli* (*E. coli*) is a pathogenic bacterium that causes infections in almost all age groups, and has been implicated in food and water contamination. Current diagnostic and detection methods tend to be labor-intensive or expensive, necessitating the need for an easy, sensitive, rapid, and low-cost method. We report on the synthesis and use of positively charged chitosan stabilized silver nanoparticles (Chi-AgNPs) as a sensitive electrochemical nanobiosensor for the detection of LPS. Chi-AgNPs were synthesized through a facile, single step protocol, and characterized for size, charge, and morphology. Glassy carbon electrodes modified with Chi-AgNPs resulted in an enhancement of signal in the presence of both LPS and *E. coli*. Detection was accomplished over a large concentration range (several orders of magnitude) of 0.001–100 ng/mL and 10–10^7^ CFU/mL. The biosensors can reliably detect LPS and *E. coli* at very low concentrations. Chi-AgNPs have potential as low cost, sensitive nanobiosensors for Gram-negative bacteria due to strong electrostatic interaction with LPS present in their outer membranes.

## 1. Introduction

Gram-negative bacteria are among the most common pathogens that affect public health, and impact environmental and food safety. These bacteria are ubiquitous, found in virtually all environments that support life. Examples include *Escherichia coli* (*E. coli*) as well as many pathogenic bacteria, such as *Pseudomonas aeruginosa* and *Yersinia pestis*. For instance, *E. coli* is one of the most widespread Gram-negative bacteria related to food-borne diseases and can cause abdominal pain, diarrhea, inflammation, or even severe cases of enteritis and hemolysis and hemorrhage, particularly in infants [1,2]. *E. coli* has been identified as an indicator of fecal contamination that can affect water supplies [3]. These important considerations make its rapid and accurate detection critical. Similarly, other pathogenic Gram-negative bacteria have been implicated in disease outbreaks and biodefense. Most conventional methods for detection involve culturing and plate counting using selective media. Though such methods are reliable and accurate, they tend to be time-consuming and laborious due to the long incubation time required for the growth of bacteria [4,5]. Nucleic-based methods such as those based on polymerase chain reaction (PCR) have been the best alternatives to culturing methods as they provide rapid identification and quantification. However, most PCR-based detection platforms may be unsuitable for resource-limited settings. Moreover, DNA typing may not be an adequate predictor of virulence [6], and also cannot efficiently differentiate between nonviable and viable cells [7,8]. Therefore, orthogonal approaches that are sensitive, fast, inexpensive, and easy-to-use for detection of Gram-negative bacteria are in urgent demand for environmental monitoring, clinical diagnosis, and food and pharmaceutical safety.

Lipopolysaccharide (LPS) is a major component of the outer membrane of Gram-negative bacteria and is an endotoxin [9,10]. Human exposure to microbial endotoxins, either by direct contact or in a systemic manner, can result in inflammatory reactions, leading to multiple organ failure, shock, and even death [11]. Increased LPS release in the body initiates an excessive innate immune response, resulting in conditions such as organ failure, septic shock, diarrhea, hypotension, vascular blood clotting, and even death [12,13]. LPS has been an important biomarker helping in the serological differentiation of Gram-negative bacteria. This, in turn, allows for the characterization and identification of pathotypes, aiding in the timely and precise treatment of infections. The development of a facile and sensitive method based on LPS sensing can therefore provide an orthogonal route for detecting Gram-negative bacteria. In this work, we use *E. coli* as a model organism to show how the detection of LPS provides a method for indirect detection. Being a virulence factor, the structure and function of LPS determine the *E. coli* serogroup, with implications for vaccine design and therapeutic interventions [9,14].

Electrochemical (bio)sensors are promising analytical tools for the detection of toxins due to their inherent advantages such as high specificity, sensitivity, cost-effectiveness, real-time application, and potential for portable instrumentation [15]. Various electrochemical sensors have been reported for the detection of LPS (summarized in Table 1 below). These methods are capable of detecting LPS at low concentrations. However, most of these sensors employ proteins, enzymes, or aptamers as ligands that are expensive, or prone to denaturing in real-world field usage. The design of a robust electrochemical sensor with a stable ligand for the sensitive detection of LPS can therefore be of great benefit for practical applications, both in terms of detection of LPS, an endotoxin in its own right, and for detection of targets such as *E. coli*.

Nanoparticle-based electrochemical signal amplification can improve both sensitivity and selectivity for electrochemical sensors and biosensors [25]. Functionalized nanoparticles not only accelerate signal transduction through synergistic catalytic activity and conductivity, but also amplify biorecognition events with specifically designed signal tags, leading to highly sensitive sensing [26,27]. Among various nanoparticle systems, silver nanoparticles (AgNPs) have gained attention due to high biocompatibility, low toxicity, and sustainable electrocatalytic activity [28]. However, increased aggregation in AgNPs due to strong van der Waals forces can result in a dramatic decline in their electrochemical activity and detection sensitivity [29,30].

In this work, we report on the use of AgNPs for the ultrasensitive detection of LPS. In order to stabilize the AgNPs and provide a positive charge, we use a low-cost bioderived material in the form of chitosan that allows the formation of robust, stable NPs. Chitosan is a biodegradable copolymer and has shown intrinsic bactericidal potential against both Gram-positive and Gram-negative bacteria [31,32]. While the exact mechanism for its antimicrobial activity has not been fully elucidated, it has been suggested that the positively charged material generates electrostatic interactions with the negatively charged bacterial cell wall leading to the leakage of intracellular constituents [33]. Chitosan has been previously reported as a stabilizing agent for AgNPs with interesting antibacterial and anti-inflammatory effects [34,35]. We show that chitosan-stabilized AgNPs (Chi-AgNPs) can be used for the electrochemical detection of negatively charged LPS and thereby *E. coli* as a model Gram-negative bacterium. As the main component of the outer membrane of *E. coli*, the negatively charged LPS can show strong electrostatic interactions with the positively charged chitosan stabilized AgNPs. Owing to these interactions, an efficient, low-cost, rapid and sensitive electrochemical nanobiosensor is rationalized for detection of LPS and *E. coli*.

## 2. Materials and Methods

### 2.1. Materials

Low molecular weight chitosan and silver nitrate were obtained from Sigma-Aldrich (St. Louis, MO, USA). Lipopolysaccharides (LPS) was purchased from Sigma-Aldrich. *E. coli* serotype O127:B8 (ATCC strain 12740) was maintained on Tryptic Soy Agar (Becton Dickinson, Franklin Lakes, NJ, USA). Prior to LPS extraction, a single colony of *E. coli* was harvested from an agar plate and inoculated into 200 mL of Tryptic Soy Broth (Becton Dickinson, Franklin Lakes, NJ, USA). The culture was grown overnight in a shaking incubator at 37 °C and 200 rpm. After incubation, the culture was centrifuged at 3000× g for 5 min and then washed twice in 1× PBS, before eluting to a final concentration of ~10^8^ CFU/mL. Concentrations were determined by serial dilutions and manual counting. In order to test the sensor, a new culture was prepared and its concentration determined by manual counting.

### 2.2. Synthesis of Chitosan Stabilized AgNPs (Chi-AgNPs)

A facile one-pot synthesis protocol was followed to synthesize Chi-AgNPs. Chitosan (0.2 g) was dissolved in 1% solution of acetic acid (10 mL) and stirred for 30 min. The resulting solution was filtered in order to remove any impurities. 100 µL of 1 M NaOH and 3 mL of 0.1 M freshly prepared AgNO_3_ were then added to the already prepared 1% acetic acid containing chitosan solution. The final solution was mixed well and stirred at 75 °C for 10 h. The color of the solution changed from colorless to light yellow and then to yellowish brown, indicating the synthesis of Ag NPs [36]. Chi-AgNPs were further authenticated by recording their surface plasmon resonance using a UV-Vis spectrophotometer (UV-240, Shimadzu, Hitachi, Japan).

### 2.3. Characterization

The synthesized Chi-AgNPs were further characterized for their size, polydispersity index (PDI), zeta potential, and surface morphology using zeta-sizer (Nano ZS90 Malvern Instruments, Malvern, UK) and Atomic Force microscope (AFM, Agilent 5500, Santa Clara, CA, USA). FT-IR analysis was used to identify the functional groups of chitosan involved in the stabilization of AgNPs using an IR spectrophotometer (Shimadzu, Kyoto, Japan).

### 2.4. Electrode Modification and Electrochemical Detection

Electrochemical measurements were conducted using a Gamry Interface 1010E Potentiostat (Gamry Instruments, Warminster, PA, USA). A conventional three-electrode system was used with a glassy carbon electrode (GCE) or a Chi-AgNP modified GCE as the working electrode, a Pt counter electrode, and Ag/AgCl as the standard reference electrode. To obtain the Chi-AgNP functionalized GCE working electrode, 8 µL of the Chi-AgNP solution was placed on the GCE electrode and dried at room temperature in a dust free atmosphere. The area of the active electrode surface is ~7 mm^2^. The stability of attachment of the NPs was verified by observing the electrode over several washing steps and extended incubation in 1× PBS buffer. Images showing the electrode surface before and after several hours of incubation with PBS are presented in the Supporting Information (Figure S1).

Cyclic voltammetry (CV) was carried out to characterize the biosensor and for electrochemical detection of both LPS and *E. coli*. Scanning was carried out from −1 to 1 V at 50 mV·s^−1^. For electrochemical analysis, a solution of LPS and suspension of *E. coli* were prepared in PBS. Bare GCE or Chi-AgNP modified GCE (sensing electrode) were immersed in PBS and their cyclic voltammograms were recorded as reference CVs for analysis. For the detection of LPS, a stock solution was prepared in 1× PBS with serial dilutions of 0.001–100 ng/mL used for further electrochemical detection. Similarly, *E. coli* serial dilutions were prepared in 1× PBS at 10–10^7^ CFU/mL from freshly grown bacteria followed by electrochemical detection. Current signals for sensing experiments were normalized to their values with respect current signals of modified electrode in the absence of any LPS or *E. coli* concentration.

## 3. Results

### 3.1. Synthesis of Chi-AgNPs

**Figure 1:**
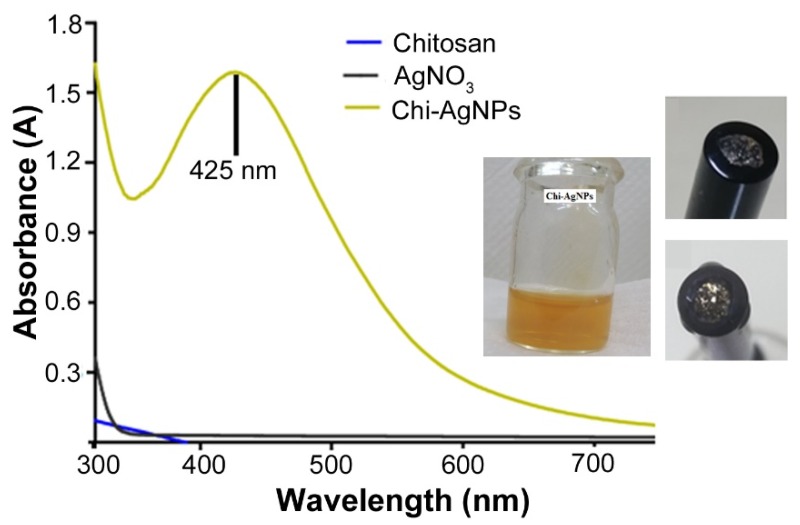
UV-vis spectral analysis of the synthesized Chi-AgNP, chitosan and AgNO_3_. The inset shows an image of the stabilized NPs and the modified electrodes. Sensing electrodes are formed by depositing 8 µl of the solution (inset) on glassy carbon. On drying, the particles are firmly adhered and do not detach even after several hours of incubation in PBS (inset).

The use of nature-derived biopolymers such as chitosan has received attention for the fabrication and stabilization of metal nanoparticles owing to biological and environmental safety and cost-effectiveness [37]. Metal nanoparticles that have been reduced/stabilized with biopolymers demonstrate higher stability against pH and electrolytes changes in comparison to their counterparts stabilized with synthetic or other small molecules [38]. In the current study, chitosan was employed as the reducing as well as a stabilizing agent for the synthesis of AgNPs. The facile, one-pot, aqueous synthesis process makes it green and scalable. UV-visible spectrophotometry is a reliable tool for confirming the synthesis of metal-based nanoparticles and was used. Chitosan and AgNO_3_ do not show characteristic peaks when screened over the range of 300–750 nm as shown in Figure 1. A single surface plasmon resonance is observed for Chi-AgNPs with peak height of 1.58 at 425 nm, which is a characteristic marker for the successful synthesis of AgNPs. The characteristic peak is narrow with good height, indicating synthesis of chitosan stabilized AgNPs with a tight size distribution.

### 3.2. Characterization of Chi-AgNPs

**Figure 2:**
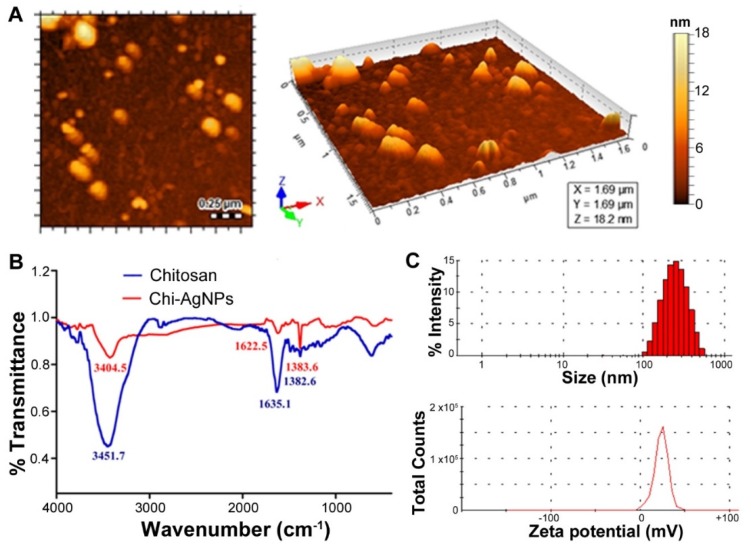
Characterization of Chi-AgNPs. (**A**) surface morphological analysis of synthesized Chi-AgNPs and (**B**) FT-IR analysis of chitosan and Chi-AgNPs; (**C**) analysis of synthesized Chi-AgNPs for size distribution and Zeta potential.

The synthesized chitosan stabilized AgNPs were then characterized for their size, polydispersity index (PDI), zeta potential, and surface morphology. AFM imaging shows the spherical morphology of the synthesized AgNPs as seen in Figure 2A. The FT-IR spectrum of chitosan reveals a characteristic peak of N-H stretching merged with O-H absorption at 3451.7 cm^−1^. Similarly, the N-H group bending vibration appears at 1635.1 cm^−1^ as shown in Figure 2B [39]. The spectrum for Chi-AgNPs shows that the N-H stretching of chitosan shifts to lower frequency of 3404.5 cm^−1^, whereas the amino group bending vibration shifts to 1622.5 cm^−1.^ These findings suggest the involvement of the amino groups of chitosan for the reduction/stabilization of AgNPs, confirming the attachment of chitosan to the nanoparticle surfaces.

Size analysis revealed that the AgNPs have an average size of 202.93 ± 4.11 nm (Figure 2C). The size lies well within the nanoscale and is therefore well suited for the construction of a nanobiosensor. The particles are monodisperse as indicated by a low PDI value of 0.22 ± 0.03. The surface charge reveals a net positive zeta potential of 23.3 ± 3.84 mV (Figure 2C). The raw data are presented in the supporting information (Figure S1, Table S1). The surface positivity of the synthesized nanoparticles can be attributed to their stabilization with the positively charged chitosan [40]. This increased surface positivity is advantageous both in terms of imparting physical stability, as well as achieving the desired sensitive sensing of the negatively charged LPS and *E. coli*. Highly positive nanoparticles repel each other preventing agglomeration over time.

### 3.3. Stability of Chi-AgNPs

**Figure 3:**
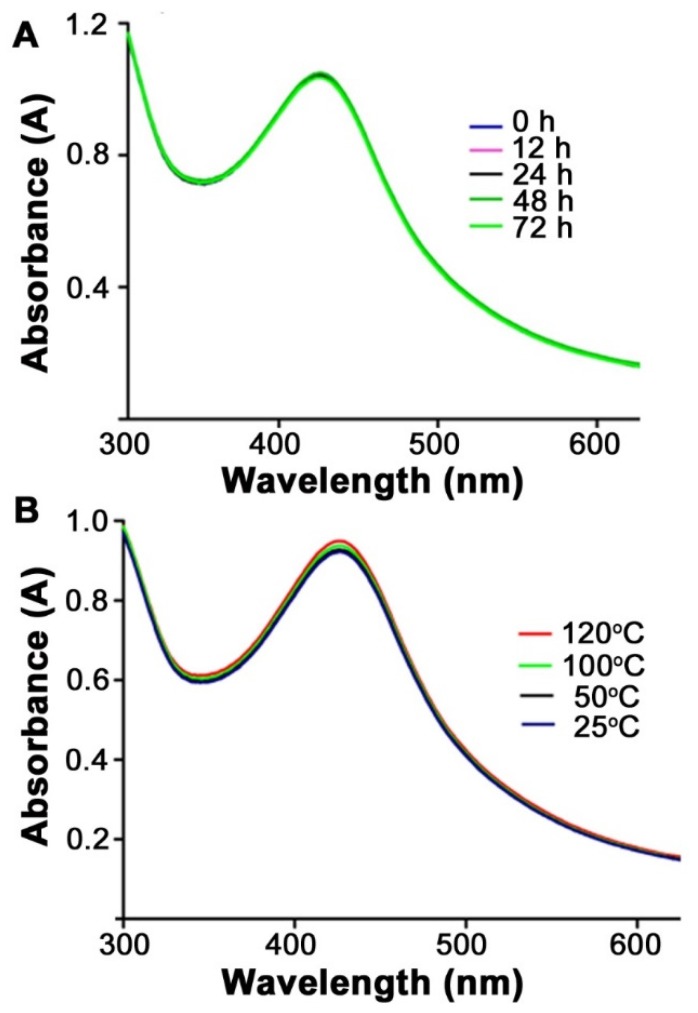
(**A**) Storage and (**B**) heat stability studies of the synthesized Chi-AgNPs.

The synthesized Chi-AgNPs were investigated for their storage stability up to 72 h. The nanoparticle solution was stored at room temperature and its characteristic surface plasmon resonance was measured through UV-vis spectrophotometry at different time intervals (Figure 3). No aggregation was observed over 72 h and the particles retained their morphology and surface characteristics. Their characteristic surface plasmon resonance did not change up to 72 h upon storage, thus demonstrating they are highly stable. In order to consider their use for a robust biosensor, their stability to high temperatures was measured. The Chi-AgNPs were heated and their characteristic surface plasmon resonance was read through UV-vis spectrophotometer over a 25–120 °C range. When subjected to heat stability, the synthesized Chi-AgNPs were found stable even up to 100 °C. Around 120 °C, their characteristic surface plasmon resonance only slightly increased without any deviation as shown in Figure 3. This provides a major advantage of using this system for biosensing in comparison to the relatively fragile and expensive aptamers, enzymes, or antibodies typically used.

### 3.4. Electrochemical Detection 

Bare GCE and Chi-AgNPs modified GCE electrodes (sensing electrode) were first screened for their characteristic CVs using PBS as the medium. The sensing electrodes are formed by deposition of 8 ul of the NP solution on the GCE (inset of Figure 1). These Chi-AgNPs are firmly adhered to the electrode and do not detach even after several washing steps or hours of incubation (Figure S2). The sample voltammograms are shown in Figure S3. The bare GCE does not show any characteristic redox peak when screened over −1 V to 1 V in PBS. In comparison, the sensing electrode reveals a characteristic peak of oxidation/reduction potential at 75.08 mV with a net current of 22.95 µA. The enhanced electrochemical behavior of Chi-AgNPs may be attributed to the ability to form constructive networks on the surface of the GCE, which in turn accelerates flow of electrons [41].

**Figure 4:**
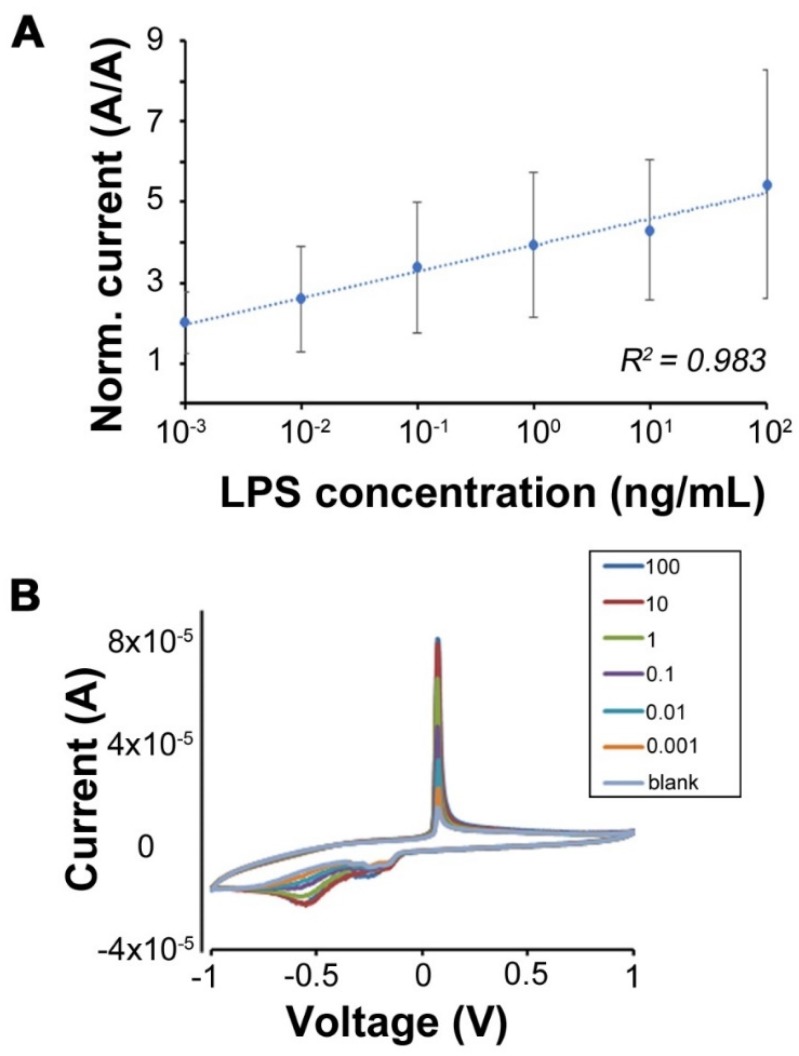
Analysis of Chi-AgNPs modified electrodes for LPS detection over 0.001–100 ng/mL. (**A**) calibration curve showing the linear response over six orders of magnitude in concentration. Here, the currents have been normalized to their value at 0 (blank). *n* = 3 different samples tested; error bars represent standard deviation for three independent experiments; (**B**) example CV showing the response to increasing LPS concentration.

The modified electrodes were first dipped in PBS until the signal was stable (there was no change in the current response of the modified electrode after 45 min; the current remained stable). The stable sensing electrodes were then used for detection of LPS at a concentration range of 0.001–100 ng/mL. The current response of the sensing electrode increased along the increasing concentration of LPS in PBS as shown in Figure 4 (Figure S4 shows the electrode response in the absence and presence of LPS). It is important to note that a steady sensor response was obtained on the order of a few minutes, which is comparable to other reports for electrochemical biosensors. A calibration curve was constructed for the tested LPS concentration against the respective current response. The line was fit with an R^2^ value of 0.98, indicating a clear linear relationship between the change in current and the tested LPS concentrations over several orders of magnitude. Based on ICH guidelines, the limits of detection (LOD) and quantitation (LOQ) were estimated from the linear regression of the calibration curve. The LOD is the lowest concentration of the analyte in a sample that can be detected, but not necessarily quantified, under the stated conditions of the test. In comparison, the LOQ is the lowest concentration of the analyte in a sample that can be determined with acceptable precision and accuracy under the stated conditions of the test [42]. The LOD of the biosensor is ~0.001 ng/mL, which is extremely competitive in comparison to recent reports on LPS biosensors [10]. The LOQ of the biosensor is ~0.003 ng/mL. The current response for 100 ng/mL further validates the efficiency of the modified electrode to detect LPS over a wide range.

The change in current response over increasing concentration of LPS can be attributed to the strong electrostatic interaction between Chi-AgNPs and LPS. This is in agreement with previously published reports where immobilized cationic peptides or proteins have been used for electrostatic recognition and ultimate electrochemical detection of LPS [11,16,17,18,20]. Moreover, the enhancement in the electrochemical signal intensity over increasing LPS concentrations is also in good agreement with previous published reports [43,44]. Overall, the sensor performance in terms of LOD and dynamic range is comparable to, or exceeds, the reported values (Table 1).

**Figure 5:**
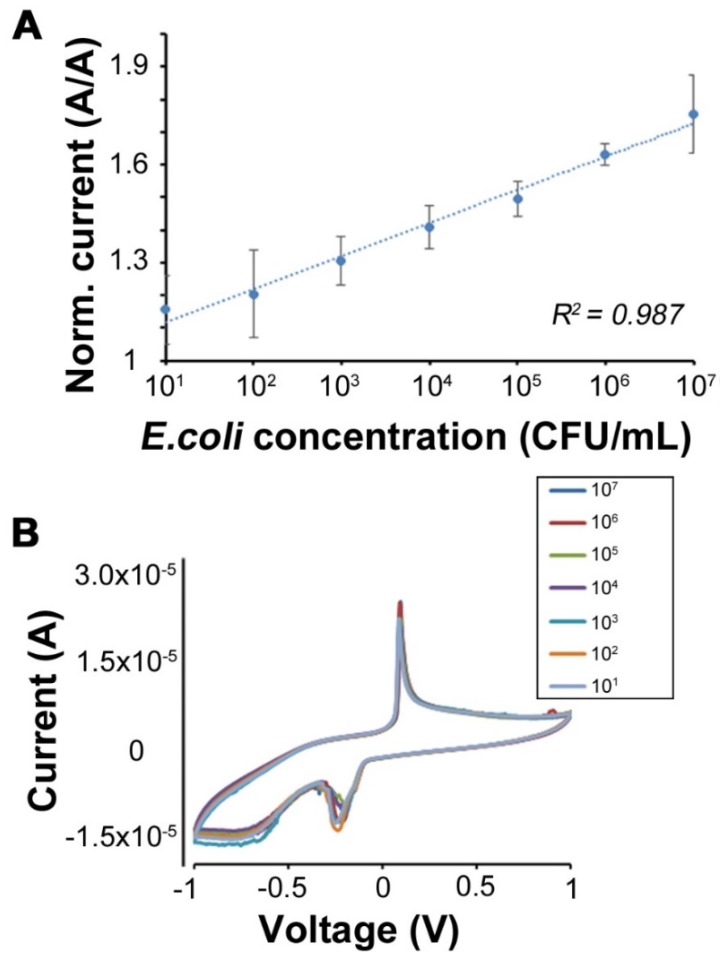
Analysis of Chi-AgNPs modified electrodes for *E. coli* detection over 10–10^7^ CFU/mL (**A**) Calibration curve showing the linear response over seven orders of magnitude in concentration. Here, the currents have been normalized to their value at 0 (blank). *n* = 3 different samples tested; error bars represent standard deviation for three independent experiments; (**B**) example CV showing the response to increasing *E. coli* concentration.

To verify if the interaction between the Chi-AgNPs modified GCE electrodes and LPS could be extended to the Gram-negative *E. coli* (as a model organism), detection was performed over a concentration range of 10–10^7^ CFU/mL. Results show that the current response of the sensing electrodes increases along the concentration of *E. coli* in PBS as shown in Figure 5. The efficiency of the fabricated sensor can be inferred from its sensing of the highest tested concentration (10^7^ CFU/mL). The constructed calibration curve further shows a clear linear relationship between the bacterial concentration and current response (over 7 orders of magnitude). The LOD for this sensor was estimated from the linear regression to be ~248 CFU/mL. The LOQ for the sensor was estimated to be ~754 CFU/mL. These values are very competitive in comparison to earlier reports [45]. LPS is a major component of the outer membrane of almost all Gram-negative bacteria. The nanoparticles used as sensing part of the detection system actively interact with the lipid A region of the LPS. Thus, they remain anchored to the bacterial surface [46]. The sensitive *E. coli* detection capabilities of the sensor can therefore be attributed to the fact that positively charged chitosan electrostatically interacts with LPS in the outer membrane of target bacteria. In the current study, Chi-AgNPs are able to achieve ultrasensitive detection of the target *E. coli* due to the presence of positively charged chitosan that establishes electrostatic interactions with LPS in the outer membrane. In all these experiments, the values are reported for three independent experiments with different sensors. The current values were normalized to the sensor current in the absence of any analyte (zero LPS or *E. coli* concentration). Finally, the utility of the sensor to detect unknown samples was performed. A suspension of *E. coli* was tested with a freshly prepared sensor and the concentration obtained using the calibration curve shown in Figure 5 (raw data presented in Figure S5). The sensor indicated a concentration of 1.31 × 10^8^ CFU/mL, which was very close to the concentration of the suspension of ~1 × 10^8^ CFU/mL, which was obtained by the standard method of counting colonies manually. Note that this is just above the dynamic range shown in Figure 5. However, this value shows the utility of the presented biosensor for the direct, online, and automated detection of bacteria such as *E. coli*.

## 4. Discussion

Lipopolysaccharides consist of a lipid (lipid A) and a core polysaccharide. One of the most common methods for LPS determination is based on the limulus amoebocyte lysate test, which is rapid and sensitive, with a detection limit of ~0.4 pg [47]. It may be noted that the extraction of bacterial lipopolysaccharides from soil samples has also been reported earlier. LPS can be extracted using an aqueous hot phenol method [48]. LPS therefore provides a method of testing for viable bacterial cells, since the LPS of dead bacteria is lost rapidly. It has been noted that there are associated problems such as quantitative extraction of lipopolysaccharide from the sample is difficult, and the specificity of this test in some cases is questionable [47]. The development of rapid and quantitative assays for LPS can be of immense benefit.

Thus, this report presents a versatile modality for the detection of the endotoxin LPS and orthogonally, the pathogen *E. coli*, which is used as a model Gram-negative organism. To the best of our knowledge, chitosan stabilized AgNPs have not been previously used for LPS detection. In a recent publication, chitosan-coated iron oxide magnetic nanoparticles were reported for the colorimetric detection of *E. coli* and the Gram-positive *Staphylococcus aureus* [49]. A similar electrostatic interaction was observed showing the universality of the nanoparticle-LPS detection. Other chitosan stabilized nanobiosensors have been reported previously for various Gram-negative bacteria [50,51]. However, these nanosensors were multifunctional and did not provide mechanism for LPS based detection of Gram-negative bacteria. In contrast, our study elucidates LPS based detection of bacteria such as *E. coli* while using a facile electrochemical setup. Furthermore, some nanoparticle systems have been used for LPS detection, but typically employ proteins, peptides, or aptamers [52,53,54]. The facile nature of the sensor fabrication provides a significant advantage in this regard. Importantly, the biosensors are not only robust at high temperatures, but also easily reusable. By simply washing the sensor with PBS followed by immersion in buffer for a few minutes, the sensors can be regenerated and reused. Thus, this system provides an inherently low cost, easy-to-use biosensor for ultrasensitive detection over a large dynamic range.

## 5. Conclusions

Pathogenic Gram-negative bacteria such as *E. coli* have been widely linked to severe issues related to public health and environmental safety. Highly sensitive, rapid, and easy to use methods are required for bacterial early detection, to replace currently used diagnostic methods that are time-consuming, expensive, and laborious. We report on positively charged chitosan-stabilized AgNPs as electrochemical biosensors for the sensitive, orthogonal detection of *E. coli*. GCE electrodes modified with the nanoscale particles are capable of ultrasensitive detection of Gram-negative bacterial owing to their electrostatic interaction with negatively charged LPS present on the outer membranes. In comparison to reported electrochemical sensors for LPS/*E. coli* that employ aptamers, proteins, or peptides as ligands that are not stable in harsh experimental conditions, this system is ultrasensitive, stable, and low cost using a renewable biopolymer. The use of the synthesized positively charged nanoparticles as sensitive nanobiosensing materials for the detection of *E. coli* can therefore be inferred.

## Figures and Tables

**Table 1:** Summary of ligands and detection in various reported electrochemical LPS sensors.

Ligand Used	Limit of Detection (LOD)	Range	Ref.
Polymyxin B	1 µg/mL	1–100 µg/mL	[16]
Positively charged peptide	1 ng/mL	1–200 ng/mL	[17]
Concanavalin A	50 µg/mL	50–200 µg/mL	[11]
Polymyxin B	0.1 μg/ml	0.1–1000 µg/mL	[18]
Cu and nitrilotriacetic acid complex	0.0001 ng/mL	0.0001–0.1 ng/mL	[19]
Antimicrobial proteins	1 ng/mL	1–100 ng/mL	[20]
Polymyxin B	0.2 ng/mL	0.2–0.8 ng/mL	[21]
Enzymes	50 ng/mL	0–10 ug/mL	[22]
Aptamer	0.001 ng/mL	0.001–1 ng/mL	[23]
Aptamer	0.01 ng/mL	0.01–1 ng/mL	[24]

## Supplementary Materials

The following are available online at https://www.mdpi.com/2072-666X/11/4/413/s1, Figure S1: Size and size distribution (polydispersity index) of the synthesized Chi-AgNPs. Figure S2: Images of electrode surfaces. Figure S3: CVs of bare and Chi-AgNPs modified electrodes in PBS. Figure S4: CVs of bare of electrodes in presence and absence of LPS. Figure S5: Measurement of unknown sample and concentration. Table S1: Size, PDI and zeta potential of the synthesized Chi-AgNPs.
