# Supplementary material for: Chitosan Stabilized Silver Nanoparticles for the Electrochemical Detection of Lipopolysaccharide: A Facile Biosensing Approach for Gram-Negative Bacteria
Source: Micromachines (Basel). 2020 Apr 14;11(4):413. doi: 10.3390/mi11040413 (PMC7231338; doi:10.3390/mi11040413)
Supplement: Supplementary file 1 [file micromachines-11-00413-s001.pdf]

Supplementary Materials: Chitosan Stabilized Silver Nanoparticles for the Electrochemical Detection of Lipopolysaccharide: A Facile Biosensing Approach for Gram-Negative Bacteria

Muhammad Imran, Christopher J. Ehrhardt, Massimo F. Bertino, Muhammad R. Shah, and Vamsi K. Yadavalli

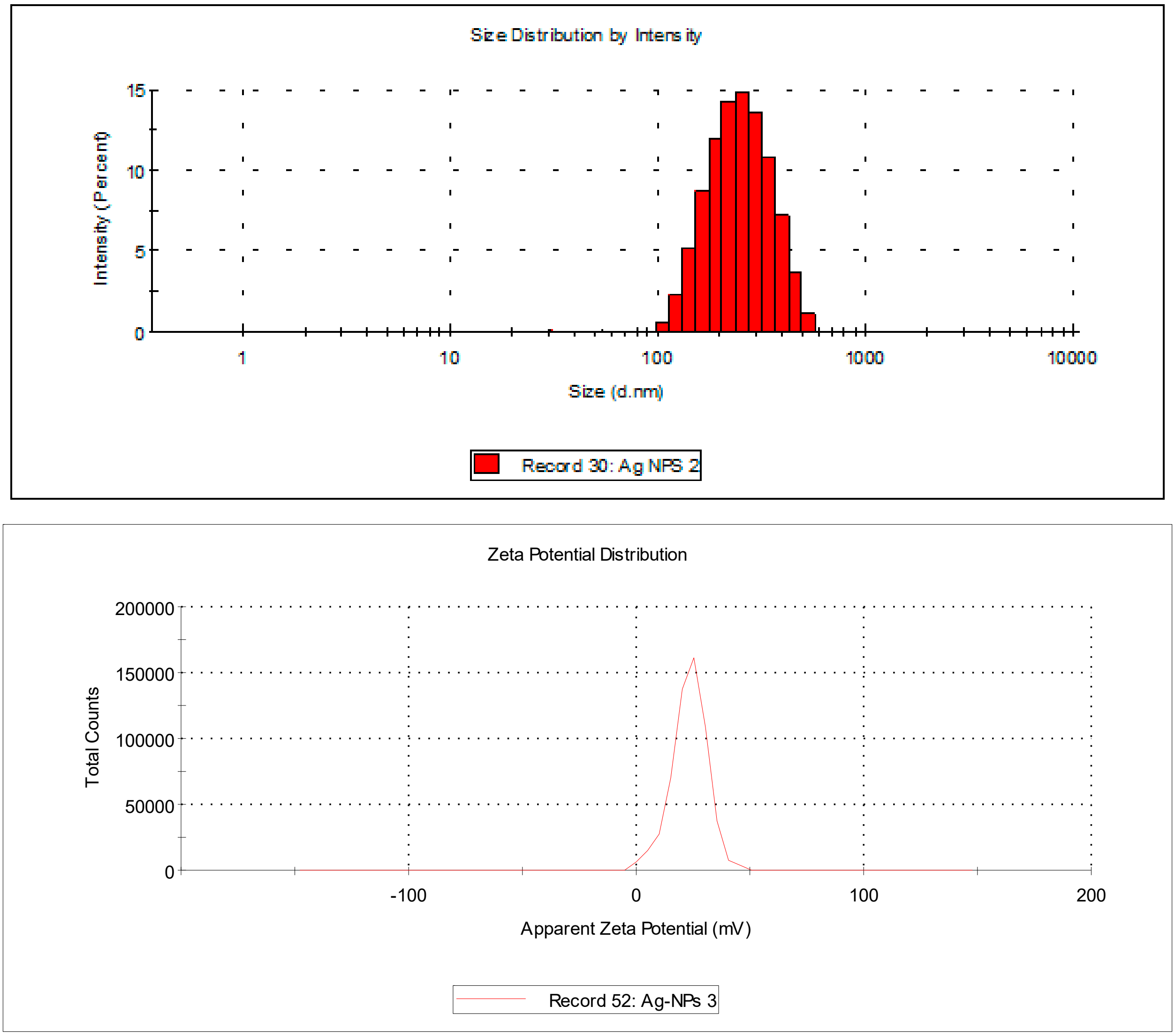

Figure S1. Particle size and size distribution (polydispersity index) of the synthesized Chi-AgNPs. Zeta potential of the synthesized Chi-AgNPs.

Table S1. Size, PDI and zeta potential of the synthesized Chi-AgNPs.

NPs	Size	PDI	Zeta Potential
Chi-Ag NPs	202.93 ± 4.11	0.22 ± 0.03	23.3 ± 3.84

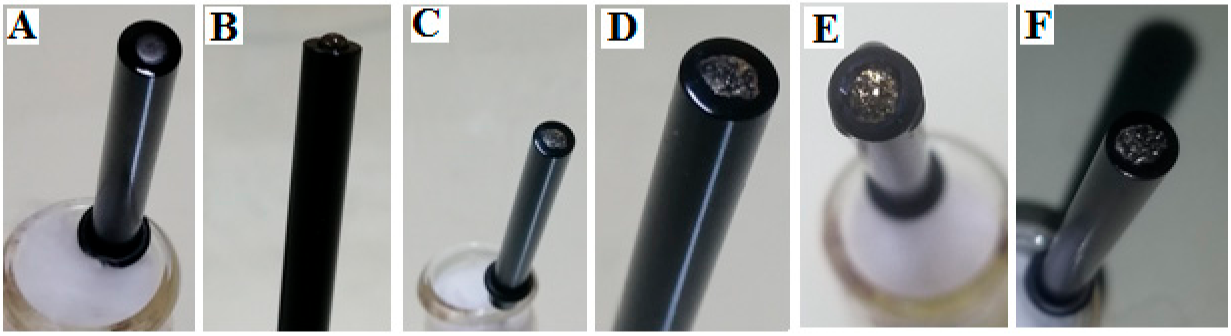

Figure S2. Images of the electrode surface (**A**) Unmodified electrode surface, (**B**) Electrode modified with Chi-AgNPS solution, (**C**) Modified electrode with coating of Chi-AgNPs, (**D**) Modified electrode after 2 h incubation with PBS, (**E**) Modified electrode after 4 h incubation with PBS and (**F**) Modified electrode after 8 h incubation with PBS. The NPs are attached to the surface and do not detach even after several hours of incubation.

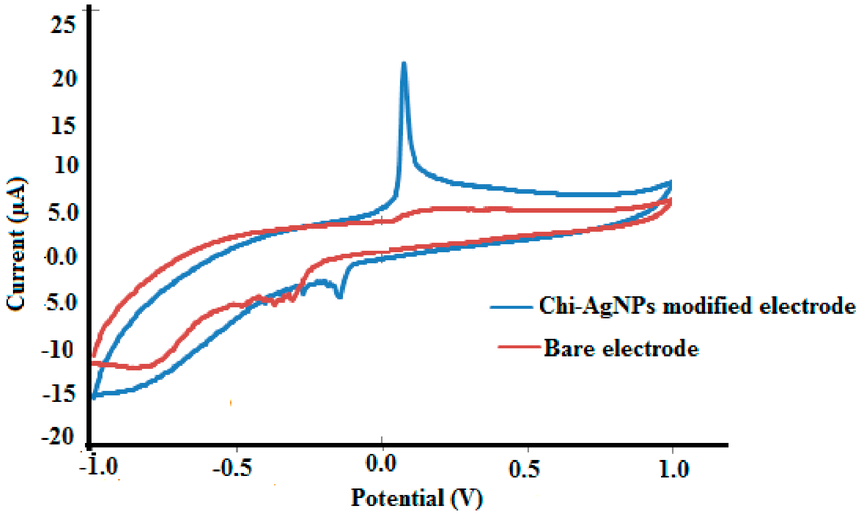

Figure S3. CVs of bare and Chi-AgNPs modified electrodes in PBS (Modified electrode shows oxidation/ reduction potential at 7.508 mV with a net current of 22.95 µA).

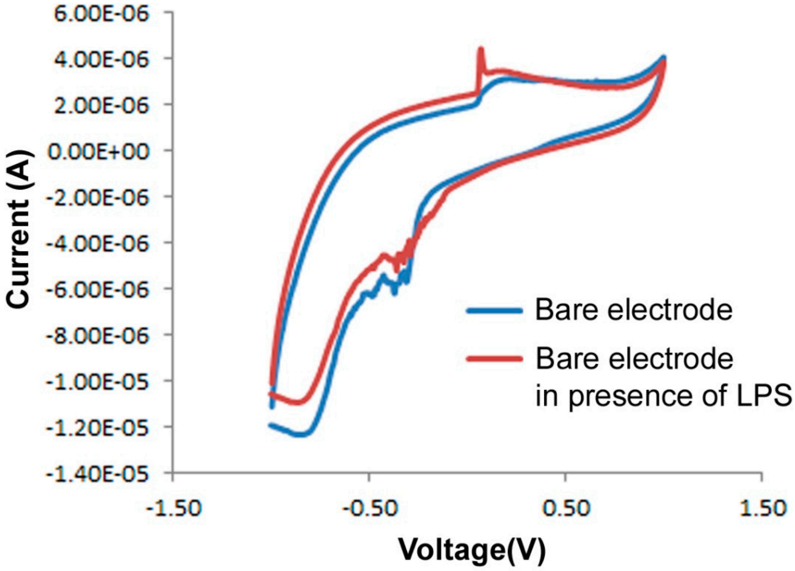

Figure S4. CVs of bare electrodes (unmodified with Chi-AgNPs) in the absence and presence of LPS. There is negligible change in signal in the presence of LPS showing that an unmodified electrode is incapable of detecting LPS or gram-negative bacteria.

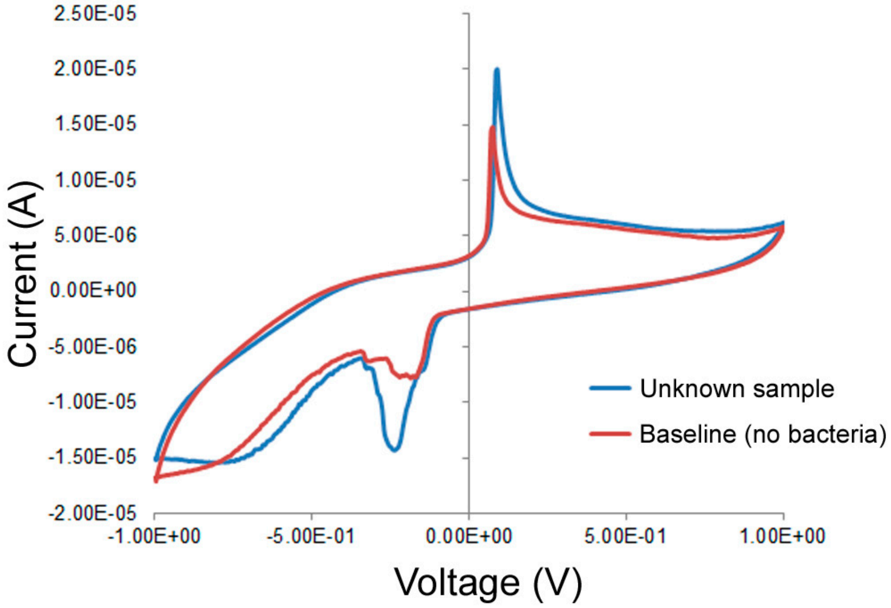

Figure S5. CVs of measurement of unknown sample and concentration obtained from calibration curve obtained in Figure 5. The calibration curve from Figure 5 has the form y = 0.0441 ln(x) + 1.0157. At the peak, value of baseline = 1.09E-05 A, value of unknown sample = 2.01E-05. Note that this experiment was conducted with a freshly prepared sensor. Therefore, the normalized current (*y*) = 1.84, implying that the unknown concentration ~1.3 × 10^8 CFU/ml. This number was verified against manual counting of the CFU/ml from the culture using dilutions. The value of the manual count was 1 × 10^8 CFU/ml.
